# Observation of rituximab as initial treatment in patients with minimal change disease- a retrospective study

**DOI:** 10.3389/fimmu.2025.1528996

**Published:** 2025-04-24

**Authors:** Sha Wang, Hua Liang, Lu-Yao Li, Miao-Miao Cheng, Fang-Yi Lv, Li Zong, Ke Zhao, Xiao-Yan Xiao, Xiang-Dong Yang

**Affiliations:** Department of Nephrology, Qilu Hospital of Shandong University, Jinan, Shandong, China

**Keywords:** rituximab, minimal change disease, glucocorticoid, Nephrotic Syndrome, remission

## Abstract

**Research background:**

This study was aimed to retrospectively investigate the efficacy and safety of rituximab (RTX) versus glucocorticoids (GC) as initial treatments for patients with minimal change disease (MCD).

**Research methods:**

Patients who were diagnosed with MCD through kidney biopsy and received RTX or GC as the initial treatment regimen were included and matched by propensity score (ratio: 1:1) based on age, sex, urine protein, and eGFR. The 2 groups each consist of 12 adult patients and 2 pediatric patients. We primarily observed the clinical remission rate at 24-week, the time to induction of remission in each group, the time to first relapse-free survival, relapse rate, as well as the changes in the urine protein-to-creatinine ratio and serum albumin levels compared to baseline during the treatment period. The incidence of adverse effects was also observed in 2 groups during the whole period.

**Research results:**

All 28 patients (100.00%) achieved clinical remission, with 22 patients (78.57%) achieving complete remission (CR) and 6 patients (21.43%) achieving partial remission (PR) at 24-week. The median time to remission was 5 (3-7) weeks. During the 24-week follow-up, the RTX group and the GC group each had 2 patients with recurrence, resulting in a relapse rate of 14.29%. Both the RTX group and the GC group had 14 patients (100%) achieve clinical remission, with 11 patients (78.57%) reaching CR and 3 patients (21.43%) achieving PR. The median time to remission in the RTX group was 5 (3-7) weeks, while in GC group, it was 5 (3-8) weeks (p=0.728). Follow-up results at 24 weeks indicated that the UPCR levels for all MCD patients decreased from an average of 8.93 (range 6.13-17.48) g/g to 0.07 (range 0.03-0.28) g/g, with no statistically significant difference between 2 groups (P=0.945). Serum albumin levels increased from 18.60 ± 7.54 g/L to 44.39 ± 4.50 g/L, with no significant intergroup difference (P=0.601). In the RTX group, patients tolerated RTX well, with only 1 case of tachycardia occurring during infusion, which resolved spontaneously after reducing the infusion rate. In the GC group, there were no severe adverse reactions reported. However, 10 patients experienced weight gain, 3 patients exhibited elevated blood glucose levels, 2 patients presented with skin striae, and 1 patient showed elevated transaminases.

**Conclusion:**

The use of RTX can effectively induce and maintain remission in MCD patients, demonstrating efficacy comparable to those treated with GC. Furthermore, the safety profile is favorable, making it a viable alternative to GC therapy. This provides a reliable initial treatment option for patients with MCD, particularly for pediatric patients.

## 1 Introduction

Minimal change disease (MCD) is a primary cause of nephrotic syndrome (NS), with clinical manifestations including edema, significant proteinuria, and hypoalbuminemia (1). Its prevalence in adults with NS is estimated to be 10% to 15%, while it is the most common pathological type of primary glomerular disease in children (2). About 70% of patients with MCD are under 5 years old and 20% to 30% of adolescents with kidney disease have MCD (3). Although MCD is generally considered to have a good prognosis, it is associated with a high rate of relapse, and younger patients exhibit a greater tendency for recurrence (4).

The KDIGO guidelines recommend glucocorticoids (GCs) as first-line therapy for adults with minimal change disease (MCD), with alternative immunosuppressants for steroid-contraindicated cases (5). For GC-dependent, resistant, or frequently relapsing patients, immunosuppressants like cyclophosphamide, calcineurin inhibitors (CNIs), or mycophenolate mofetil (MMF) are advised (6). However, prolonged GC use increases risks of Cushing’s syndrome, osteoporosis, and growth impairment in children (7). Long-term CNIs may also cause gastrointestinal toxicity and nephrotoxicity (8).

There have been many recent advancements in the mechanics of MCD. Previous evidence supports that T cell dysregulation can lead to podocyte dysfunction, which may be a major cause of MCD (9). However, recent studies have confirmed that B cells also play a significant role in the pathogenesis of MCD. The discovery of anti-nephrin antibodies, along with the positive therapeutic effects of B cell depletion therapy, has demonstrated the important role of B cells in the pathogenesis of MCD (10).

Rituximab (RTX) is a chimeric monoclonal antibody that selectively binds to CD20 and depletes B lymphocytes, which has been approved for the treatment of various immune-mediated renal diseases, such as membranous nephropathy (11). In the treatment of MCD, the KDIGO guidelines recommend RTX as a second-line therapy for patients who are resistant to GC, dependent on GC, or cannot tolerate GC (5). Considering the adverse effects associated with long-term GC use, we designed a study to compare RTX monotherapy with traditional GC treatment in the initial management of MCD, in order to explore the feasibility of RTX as a first-line treatment for MCD.

## 2 Materials and methods

### 2.1 Patients

This study included 28 patients with MCD who were admitted to the Nephrology Department of Qilu Hospital, Shandong University, from January 2021 to December 2023.Inclusion criteria: ① Patients diagnosed with MCD through percutaneous kidney biopsy and electron microscopy. ② Biochemical levels prior to treatment meeting the diagnostic criteria for nephrotic syndrome, including nephrotic-range proteinuria (>3.5 g/d), hypoalbuminemia (plasma albumin <30 g/L), edema, and hyperlipidemia. ③ Availability of comprehensive clinical data, including blood routine, liver function, kidney function, urine protein-to-creatinine ratio, and lymphocyte subset counts. ④ Follow-up duration of at least 24 weeks. Exclusion criteria: ① Secondary kidney diseases due to various reasons, such as congenital or hereditary conditions, early connective tissue diseases, infections, or drug-induced nephropathy. ②Presence of severe comorbidities, including malignancies, infections, severe cardiovascular and cerebrovascular diseases, osteoporosis, and gastrointestinal bleeding. ③ Pregnant or lactating women. ④ Incomplete medical records or those lost to follow-up for various reasons. This study was approved by the Ethics Committee of Qilu Hospital, Shandong University.

Patients were divided into two groups based on different treatment protocols. Th RTX direct induction treatment group, which included 14 patients, of whom 8 received RTX alone and 6 received RTX combined short-term GC therapy due to very low serum albumin level. There were 12 adult patients and 2 pediatric patients. The steroid treatment group, also comprised 14 patients, with 12 adults and 2 pediatric patients.

### 2.2 Treatment methods

In the RTX group, the dosage of RTX exhibits individual variability, with a standard administration protocol consisting of either a weekly intravenous injection of 375 mg/m² for four weeks or two intravenous injections of 1 g of RTX, with a two-week interval between the injections. Alternative regimens include 1 g, 375 mg/m² ×1 dosages or 375 mg/m² ×2 dosages. Six patients with ALB below 20 g/L received full-dose corticosteroid combination therapy (1 mg/kg, continuously for 2 weeks). Following the completion of the initial RTX induction treatment, the steroid dosage was rapidly reduced and subsequently discontinued (the dose was halved after the initial RTX induction treatment, followed by a rapid taper until complete cessation). The total duration of steroid treatment was 4 weeks. The GC group received an adequate glucocorticoid regimen: prednisone or prednisolone at a dose of 1 mg/kg/day (maximum 60 mg/day), maintained for at least 4 weeks and up to 16 weeks. After alleviation, gradually reduce the dosage over a period of at least 24 weeks.

### 2.3 Data collection

Retrospective collection of baseline clinical data and patient characteristics was performed using the medical record system. The estimated glomerular filtration rate (eGFR) was calculated using the CKD-EPI formula. Baseline was defined as the first administration of RTX or GC following the diagnosis of MCD. Clinical data were collected at 4, 12 and 24 weeks post-initial induction therapy, with monitored parameters including complete blood count, urinalysis, liver and kidney function, lipid and glucose levels, 24-hour urine protein quantification, and B cell counts. Adverse events related to RTX and GC were recorded during the infusion period and throughout the entire follow-up period.

### 2.4 Definition of remissions

Time to Remission: The duration from the first RTX injection or the initial use of glucocorticoids (GC) to the time when urinary protein turns negative. Complete Remission(CR): Defined as a urinary protein-to-creatinine ratio (UPCR) of <300 mg/g, serum albumin levels of ≥35g/L, with no deterioration or progression of renal function. Partial Remission(PR): Characterized by UPCR levels between 300 mg/g and 3500 mg/g, with a reduction of at least 50% from peak levels, alongside normal or elevated serum albumin levels, or stable serum creatinine levels, defined as an increase of less than 30% from baseline. Patients who do not meet the above criteria are classified as non-responders. Relapse is defined as a return of urinary protein >3.5 g/d or UPCR >3500 mg/g (or >350 mg/mmol) after a period of partial or complete remission. Frequent Relapses: Defined as two or more relapses within 6 months (or four or more relapses within 12 months). Acute Kidney Injury (AKI): Defined as an increase in serum creatinine to ≥0.3 mg/dl within 48 hours, or an increase of ≥50% from baseline within 7 days, or a reduction in urine output [<0.5 ml/(kg/h)] lasting ≥6 hours.

### 2.5 Statistical analysis

Data were analyzed using the statistical software SPSS 27.0. Categorical variables were described using frequencies and percentages, with comparisons made using the chi-square test or Fisher’s exact probability method. Continuous variables were first assessed for normality using the Shapiro-Wilk test. Variables that met the criteria for normal distribution were expressed as mean ± standard error and group differences were evaluated using independent samples t-test or paired samples t-test. For variables that did not meet normality assumptions, medians (interquartile ranges) were reported, and differences between groups were assessed using the Mann-Whitney rank sum test or Wilcoxon signed-rank test. A p-value of <0.05 was considered statistically significant.

## 3 Results

### 3.1 Baseline data

A total of 28 patients with MCD were included in the study, with 14 patients receiving rituximab treatment (RTX group), of whom 6 were temporarily treated with steroids due to low serum albumin levels(<20g/L). The remaining 14 patients received steroid treatment (GC group). The average ages of RTX and GC groups were 34.43 ± 16.23 years and 37.43 ± 17.56 years, respectively (p = 0.643). At baseline, the median urine protein-to-creatinine ratio (UPCR) was 14.94 (5.47-19.74) vs 7.99 (6.33-13.14) (p = 0.462); the mean serum albumin was 18.66 ± 10.60 vs 18.54 ± 2.39 (p = 0.968); the median serum creatinine was 41 (20.85-60.5) vs 98 (70-133.75) (p < 0.001); and the mean estimated glomerular filtration rate (eGFR) was 120.88 ± 38.29 vs 79.75 ± 42.66 (p = 0.013) in the RTX and GC groups, respectively (**Table 1**).

**Table 1:** Baseline characteristics of patients with MCD included in the study.

Patients (n)	Total (n=28)	RTX (n=14)	GC (n=14)
Male/Female (n)	15/13	7/7	8/6
Age (years)	35.93 ± 16.66	34.43 ± 16.23	37.43 ± 17.56
Hypertension, n (%)	3 (21.4%)	2 (14.3%)	1 (7.1%)
Diabetes mellitus, n (%)	0	0	0
History (months)	0.5 (0.5-1)	1.0 (0.5-1.0)	0.5 (0.5-0.625)
UPCR (g/g)	8.93 (6.13-17.48)	14.94 (5.47-19.74)	7.99 (6.33-13.14)
Albumin (g/L)	18.60 ± 7.54	18.66 ± 10.60	18.54 ± 2.39
Serum creatinine (umol/L)	66 (38-102.25)	41 (20.85-60.5)	98 (70-133.75)
eGFR (ml/min/1.73 m^2^)	100.31 ± 44.95	120.88 ± 38.29	79.75 ± 42.66
eGFR>60, n (%)	20 (71.4%)	11 (78.6%)	9 (64.3%)
eGFR30-60, n (%)	5 (17.9%)	3 (21.4%)	2 (14.3%)
eGFR<30, n (%)	3 (21.4%)	0 (0.0%)	3 (21.4%)
RTX regimen			
4 × 375 mg/m2, once weekly	4 (28.6%)	4 (28.6%)	
2 × 375 mg/m2, once weekly	2 (14.3%)	2 (14.3%)	
2 × 1 g, every 2 weeks	6 (42.9%)	6 (42.9%)	
1 × 375 mg/m2	1 (7.1%)	1 (7.1%)	
1 × 1 g	1 (7.1%)	1 (7.1%)	

### 3.2 Analysis of the efficacy and follow-up data after treatment

We primarily observed the clinical remission rate at 24-week, the time to induction of remission in each group, the time to first relapse-free survival, and relapse rate. The time to first relapse-free survival is defined as the duration from the initial rituximab treatment to the first relapse. A total of 28 patients with MCD were followed for 24 weeks. During the treatment period, all 28 patients (100.00%) achieved clinical remission, with 22 patients (78.57%) achieving CR and 6 patients (21.43%) achieving PR. The median time to remission was 5(3 – 7) weeks. Within the 24-week follow-up, 4 patients relapsed, resulting in a relapse rate of 14.29%. Both RTX group and GC group had 14 patients (100%) achieving clinical remission, with 11 patients (78.57%) achieving CR and 3 patients (21.43%) achieving PR. The median time to remission in the RTX group was 5(3 - 7)weeks, while in the GC group it was 5(3 - 8)weeks. In the RTX group, 1 patient relapsed during the 24-week follow-up, with a relapse-free survival time of 24 weeks, and subsequently received prednisone at 30 mg/day, resulting in renewed remission. In GC group, 3 patients relapsed during the follow-up period, with relapse-free survival times of 12 weeks, 12 weeks, and 20 weeks, primarily associated with steroid tapering. (**Table 2**).

**Table 2:** Remission and relapse in patients.

**Characteristic**	**Total(n=28)**	**RTX(n=14)**	**GC(n=14)**	**P**
Time to achieve remission (weeks)	5 (3-7)	5 (3-7)	5 (3-8)	0.728
Clinical remission, n (%)	28 (100%)	14 (100%)	14 (100%)	–
CR, n (%)	22 (78.57%)	11 (78.57%)	11 (78.57%)	–
PR, n (%)	6 (21.43%)	3 (21.43%)	3 (21.43%)	–
Relapse, n (%)	4 (14.29%)	1 (7.14%)	3 (21.43%)	0.596

In RTX group, 6 patients with ALB below 20 g/L received full-dose corticosteroid combination therapy (1 mg/kg, continuously for 2 weeks). All 6 patients (100.00%) in the RTX group achieved complete remission during follow-up, with a median time to remission of 4 (1.75–7.25) weeks and a median relapse-free survival time of 34 (20–39) weeks.

We also found that 10 patients were followed for 48 weeks, with only 3 relapses occurring at the 32nd and 36th week, respectively; 4 patients were followed for 96 weeks, with 2 relapses during that period, also at the 32nd and 36th week, all of which were treated with 500 mg rituximab, achieving remission, and no further relapses occurred during the 72-week follow-up among RTX group. In the GC group, 5 patients were followed for 48 weeks, with 2 relapses at the 20th and 48th week, respectively. Both relapses were managed with glucocorticoid therapy and tacrolimus, leading to remission, and no further relapses were observed subsequently. Additionally, 1 patient was followed for 96 weeks, with 1 relapse occurring at the 48th week. This relapse was treated with tacrolimus, resulting in remission, and no subsequent relapses were reported. During follow-up, B-cell count recovery was observed in 5 patients, with a mean recovery time of 38.4 ± 19.72 weeks and a mean B-cell count of 46 ± 24/μL. Among these, 4 patients maintained remission throughout the follow-up period, while one patient experienced B-cell count recovery (20/μL) at 20 weeks of follow-up, accompanied by disease relapse.

### 3.3 Comparison of laboratory indicators between two groups after treatment

**Figure 1:**
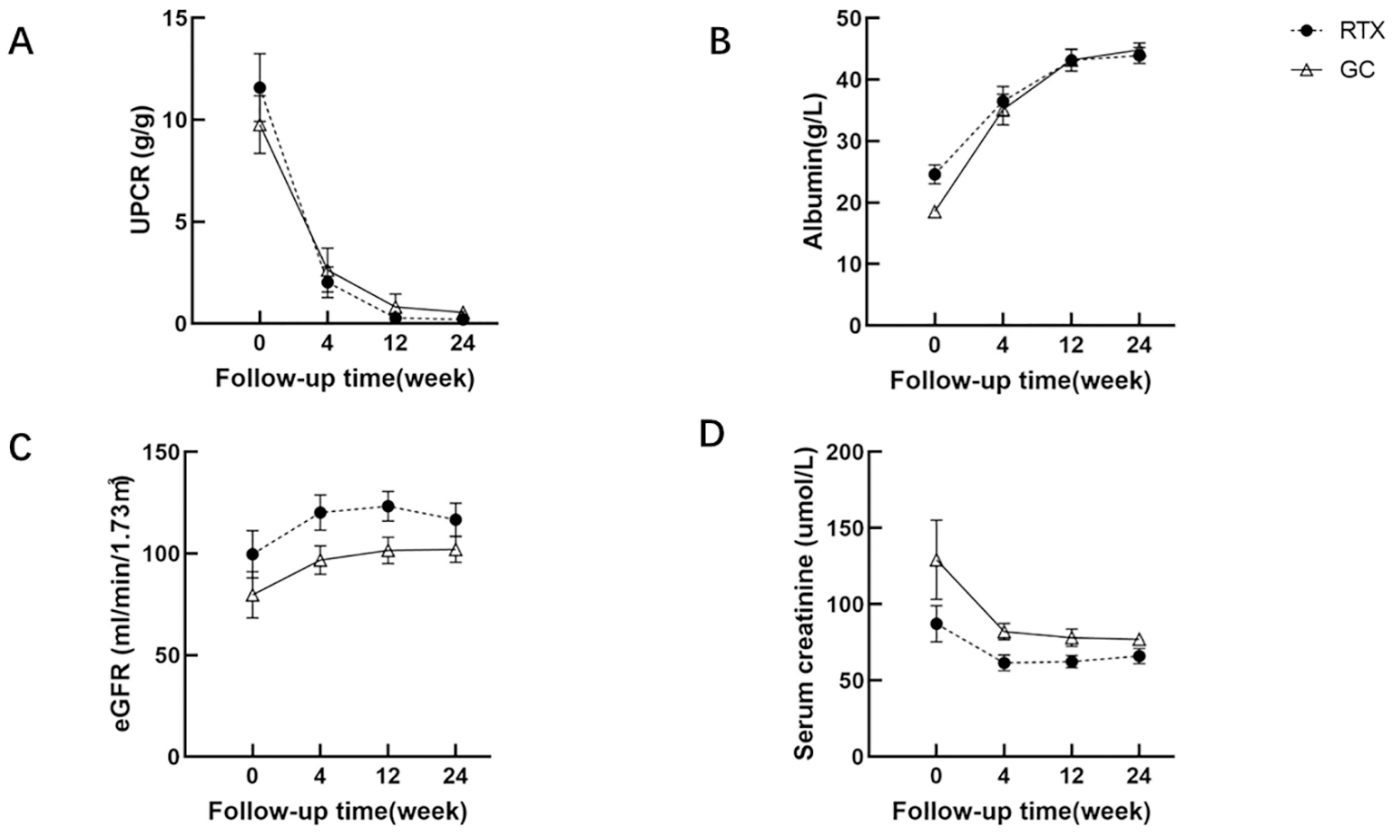
Comparison of UPCR **(A)**, serum albumin **(B)**, eGFR **(C)** and serum creatinine **(D)** between RTX group and GC group.

The follow-up results indicated that the UPCR levels of all MCD patients decreased from an median of 8.93 (6.13-17.48) g/g to 0.07 (0.03-0.28) g/g, with no statistically significant difference between the groups after treatment (P=0.945). Serum albumin levels increased from 18.60 ± 7.54 g/L to 44.39 ± 4.50 g/L, with no statistically significant difference observed between the groups after treatment (P=0.601) (**Figure 1**).

### 3.4 Adverse effects

In the RTX group, almost all patients tolerated RTX well. Dexamethasone was administered prior to infusion, and no serious adverse events were observed during the infusion for any patient. One patient experienced tachycardia during the infusion, which resolved spontaneously after reducing the infusion rate. In this study, MCD patients receiving RTX treatment did not experience adverse reactions such as granulocytopenia, cytopenia, hypertension, or gastrointestinal discomfort. In the GC group, there were no severe adverse reactions reported. Among the patients, 10 experienced weight gain, 3 exhibited elevated blood glucose levels, 2 presented with striae, and 1 showed hepatic dysfunction.

## 4 Discussion

At the 24-week follow-up of our study, we observed that the efficacy of the RTX group was comparable to that of the GC group, and both groups demonstrated favorable therapeutic outcomes. After treatment, the UPCR level in all MCD patients decreased from an average of 8.93 (range 6.13 to 17.48) g/g to 0.07 (range 0.03 to 0.28) g/g, and serum albumin increased from (18.60 ± 7.54) g/L to (44.39 ± 4.50) g/L. No statistically significant differences were observed between the groups. The RTX group showed an overall remission rate of 100%, including a complete remission (CR) rate of 78.57% and a partial remission (PR) rate of 21.43%. Additionally, in the RTX group, 5 patients had no recurrence during follow-up for over one year, and their kidney function remained stable. Their follow-up durations were 51, 74, 102, 111, and 57 weeks, respectively.

Currently, the application of RTX in MCD primarily involves two categories of patients: the first category includes those who are GC dependent, GC resistant, and frequent relapse; the second category comprises patients who cannot tolerate GC due to severe adverse reactions or who are unwilling to use GC (12). In these patients, the use of RTX can significantly help reduce GC consumption, thereby decreasing the incidence of adverse effects. A meta-analysis that included 11 studies on RTX treatment for MCD patients (n = 170) indicated an overall remission rate of 80.3% (95% CI, 68.5-88.5%) and a relapse rate of 35.9% (95% CI, 25.1-48.4%), with 74.7% achieving complete remission (13). The incidence of serious adverse events was reported as 0.092 events per year, highlighting its favorable efficacy and safety profile. However, reports of RTX as an initial treatment option for patients with GC dependent, resistant and frequent relapse. Nan Guan et al. reported on 9 cases of MCD patients who were treated with RTX as the initial regimen; among them, 5 patients received a single dose of RTX (375 mg/m²) and achieved complete remission after a median follow-up of 24 days (range, 12–48 days), with no relapses observed during the follow-up period of 303 to 884 days (14). A patient with an 11-year history of diabetes achieved partial remission after receiving a single dose of RTX, and the UPCR further decreased to 698.8 mg/g following a second dose of RTX. A patient experienced partial remission after a single infusion of RTX but soon relapsed, while two patients exhibited resistance to RTX. Notably, these three patients subsequently responded well to GC treatment, which may be related to the relatively low dose of RTX used (14).

In our study, we conducted the first retrospective analysis of this cohort, which included 28 patients newly diagnosed with MCD. Patients who were diagnosed with MCD through kidney biopsy and received RTX or GC as the initial treatment regimen were included and matched by propensity score (ratio: 1:1) based on age, sex, urine protein, and eGFR. The 2 groups each consist of 12 adult patients and 2 pediatric patients. The dosage of RTX exhibits individual variability. After excluding the presence of infections, an individualized RTX treatment plan was carried out based on the patient’s weight, general condition (such as IgG levels and presence of underlying diseases), and different age stages (minimum 16 years, maximum 75 years). The standard administration protocol involves intravenous infusion of 375 mg/m² RTX weekly for four weeks or two doses of 1 g RTX two weeks apart; alternative regimens of 1 g or 375 mg/m² are also employed. Within the RTX group, 8 patients received rituximab as monotherapy. Six patients were treated with RTX in conjunction with a short course of GC due to their low serum albumin levels, which may lead to adverse consequences such as infections, edema and further deterioration of renal function (15).

In the 6 patients receiving combined treatment with RTX and GC, we observed that this treatment not only rapidly reduced proteinuria and increased serum albumin levels but also maintained a stable remission effect during the rapid tapering of steroids. This approach significantly reduces the overall dosage of GC and the frequency of MCD relapses, while also decreasing the subsequent need for immunosuppressants. It is worth mentioning that although some patients’ B cell counts gradually returned, RTX was not re-administered, and no relapses were observed during the long-term follow-up. These results indicated that various individualized RTX dosing protocols achieved good induction remission outcomes. During the treatment period of four to six months, additional RTX doses may be appropriately administered based on the individualized treatment plan or the presence of relapse. Therefore, the findings of this study further suggest that individualized dosing of RTX, whether used alone or in combination with short-term GC therapy, may represent a novel initial induction regimen for patients with MCD.

Over the past decade, RTX has also been widely used in children with MCD (16). However, there is a lack of published literature regarding clinical experience with initial RTX treatment. F.E. Hengel and colleagues found that circulating anti-nephrin autoantibodies are prevalent among children with MCD; in a study of 182 children with idiopathic nephrotic syndrome, 94 (52%) had detectable anti-nephrin autoantibodies, which were strongly correlated with the urinary albumin-to-creatinine ratio (10). This finding is particularly significant for pediatric patients who typically do not undergo kidney biopsies. Given the unique characteristics of pediatric patients and the array of side effects associated with steroid therapy, we present here our initial experience with RTX as first-line treatment for two cases of pediatric MCD. In one case, the patient received RTX at a dosage of 375 mg/m² for induction of remission, achieving remission within one month; this patient subsequently received two additional doses of 375 mg/m² RTX for consolidation treatment without experiencing any relapse thereafter. In the second case, the patient was treated with 500 mg RTX in conjunction with short-term glucocorticoid therapy, achieving rapid remission but experiencing a relapse at the 24-week follow-up, which we suspect may be related to insufficient RTX dosing. Overall, the two cases reported in this study demonstrate successful remission using RTX as an initial treatment strategy. KDIGO 2021 guidelines recommend glucocorticoids as the first-line treatment for pediatric MCD. However, our study suggests that RTX, whether used alone or in combination with glucocorticoids, could represent a novel initial induction regimen especially for pediatric patients with MCD, potentially maintaining long-term remission while significantly reducing the side effects of glucocorticoids. However, the optimal RTX dosage should be individualized.

In this study, the vast majority of patients in the observation group tolerated RTX well, with only one patient experiencing a mild infusion reaction, which improved with symptomatic treatment. Many studies indicate that RTX is well-tolerated, with few adverse reactions (17, 18). Other research has reported that the incidence of serious adverse events can range from 0% to 17%, with infusion reactions being the most common adverse effect, often alleviated by adjusting the infusion rate (19).

However, this study has several limitations. First, it is a retrospective study with a limited sample size, which may not address all questions; further exploration through larger cohort or randomized controlled trials is needed. Second, the follow-up period for patients was relatively short, necessitating additional cohort studies with extended follow-up to confirm findings. Despite these limitations, our research remains significant, providing new insights and methods for treating newly diagnosed MCD patients, as well as offering new strategies for maintaining remission. Overall, this study provides important data to support future large-scale prospective research.

Conclusion: Both RTX and RTX combined with short-term GC effectively induce and maintain remission in MCD, demonstrating comparable efficacy to GC treatment with good safety profiles. These regimens may serve as reliable alternatives for initial treatment in MCD patients. Further research is required, particularly regarding the induction protocols and dosing of RTX in pediatric MCD patients.

## Data Availability

The original contributions presented in the study are included in the article/supplementary material. Further inquiries can be directed to the corresponding authors.
